# An Investigation of the Relationship Between Vascular Markers and Cognitive Functions in Early Hypertension

**DOI:** 10.3390/jpm14121136

**Published:** 2024-12-03

**Authors:** Réka Majer, Attila Nagy, Enikő Csikai, Mónika Andrejkovics, Ágnes Diószegi, Attila Tóth, László Csiba

**Affiliations:** 1 Department of Neurology, Faculty of Medicine, University of Debrecen, 4032 Debrecen, Hungary; 2 Clinical Psychology Center, Clinical Center, University of Debrecen, 4032 Debrecen, Hungary; 3 Faculty of Health Sciences, University of Debrecen, 4032 Debrecen, Hungary; 4 Faculty of General Medicine, Institute of Behavioural Sciences, Doctoral School of Health Sciences, University of Debrecen, 4032 Debrecen, Hungaryandrejkovics.monika@sph.unideb.hu (M.A.); 5 Department of Medicine, Faculty of Medicine, University of Debrecen, 4032 Debrecen, Hungary; 6 Faculty of Medicine, Division of Clinical Physiology, University of Debrecen, 4032 Debrecen, Hungary; atitoth@med.unideb.hu

**Keywords:** hypertension, cognitive function, fmd, imt, aix, pwv

## Abstract

**Background/Objectives**: Controlling hypertension may reduce the risk of cognitive impairment. A marker for the identification of hypertensive patients who are more likely to suffer cognitive impairment would be of clinical benefit. In our research, 105 patients with newly diagnosed primary hypertension were assessed at the Department of Neurology, the University of Debrecen. **Methods**: The available data covered detailed medical history and data, the results of different tests, ambulatory blood pressure monitoring, the intima–media thickness, the flow-mediated dilatation, the augmentation index, the pulse wave velocity, and neuropsychological evaluation. Multiple linear regression models were created to evaluate the associations found in simple analyses (Spearman’s rank correlation and Pearson’s chi-squared test). **Results**: The flow-mediated dilatation showed significant correlations with working memory, attention, learning, and executive functions. The intima–media thickness showed significant correlations with attention and reaction time. The composite flow-mediated dilatation/IMT ratio showed a significant relationship with the overall index of cognitive functions. Based on our results, a flow-mediated dilatation/IMT ratio of 15 represents a cut-off value. The pulse wave velocity showed a significant correlation with working memory and attention. The augmentation index showed significant relationships with reaction time and executive functions. **Conclusions**: Based on our results, the intima–media thickness, flow-mediated dilatation, and their ratio are suitable for the identification of a particularly vulnerable subgroup of patients for whom a detailed cognitive examination is required for the early detection and treatment of functional disorders. The assessment of attention, executive functions, working memory, and reaction time is required in early hypertension.

## 1. Introduction

Hypertension is increasingly recognized as an important risk indicator for cognitive decline and impairment in a variety of populations. The connections between hypertension and cognitive function are complex, with data indicating that both the duration and severity of hypertension can impair cognition.

Several studies demonstrate that chronic hypertension causes structural modifications in the brain, possibly contributing to cognitive impairment. For example, hypertension can cause microvascular damage, resulting in lower cerebral blood flow and subsequent cognitive impairment [1]. Furthermore, hypertension has been linked to abnormalities in brain connections, particularly in memory-critical regions like the hippocampus [2]. These structural changes can manifest as difficulties in memory, executive function, and overall cognitive performance, even in individuals with a high level of education [3,4].

The point in time of hypertension’s beginning appears to be essential. Evidence suggests that midlife hypertension poses a greater risk of cognitive decline in later life than hypertension that develops in older age [5,6]. This is particularly crucial because untreated hypertension over decades may lead to cognitive impairment, showing that early detection and therapy are critical [5]. A systematic analysis revealed that persistent hypertension throughout midlife significantly corresponds with subsequent cognitive impairment, whereas the influence of hypertension in the elderly age is less pronounced [6]. The evidence strongly supports the notion that hypertension is a modifiable risk factor for cognitive decline, particularly when it occurs in midlife.

Chronic hypertension causes the thickening of the carotid intima and media (IMT) [7]. Another ultrasound-measurable parameter is flow-mediated dilatation (FMD) in the brachial artery [8]. Abnormal arterial stiffness can also be diagnosed non-invasively with arteriography [9]. Several studies have shown that hypertension (and its associated medications) also affects cognitive performance [10,11]. In addition to reducing the risk of stroke, treating hypertension can also reduce the risk of cognitive impairment [12,13].

However, assessing cognitive functions is time-consuming. A complete, detailed cognitive assessment usually takes 1–1.5 h, including a consultation stage and an evaluation of approximately the same duration. Therefore, it is important to choose the relevant cognitive domain and examine it specifically. Since hypertensive patients make up 25–35% of the population [14], a marker is needed to select hypertensive patients who are more likely to suffer cognitive impairment. The carotid IMT, arteriography, and FMD are rapid examinations suitable for screening (duration: 6–8 min); therefore, we compared the results of these tests with neuropsychological examinations in our search for the most sensitive screening method, a threshold, and the cognitive domain with the earliest impairment.

## 2. Materials and Methods

Our research was a single-center, retrospective study that took place in the Department of Neurology, the Clinical Center of the University of Debrecen. This study was performed between January 2016 and December 2019. The study sample was produced in the following manner. We contacted occupational health doctors who regularly see professional firefighters and police officers for necessary annual medical check-ups. Those who had high blood pressure during the examination, as determined by the University of Debrecen Cardiovascular Center. Here, ABPM was used in this case, and they were chosen for the study after considering the exclusion criteria.

Based on the study protocol, we included patients presenting at their primary care provider with newly diagnosed primary hypertension (ICD code: I10). GPs and occupational health physicians aided the patient enrollment process. Asymptomatic and untreated patients whose hypertension was confirmed by ABPM and who had not yet received antihypertensive treatment were included. Primary hypertension was diagnosed following the ESC guidelines, requiring an average blood pressure exceeding 140/90 mmhg based on ABPM results. All subjects were asymptomatic and predominantly middle-aged (active age), identified by screening. Following the ABPM, a CT scan was also performed to detect asymptomatic abnormalities (e.g., silent infarction).

**Figure 1:**
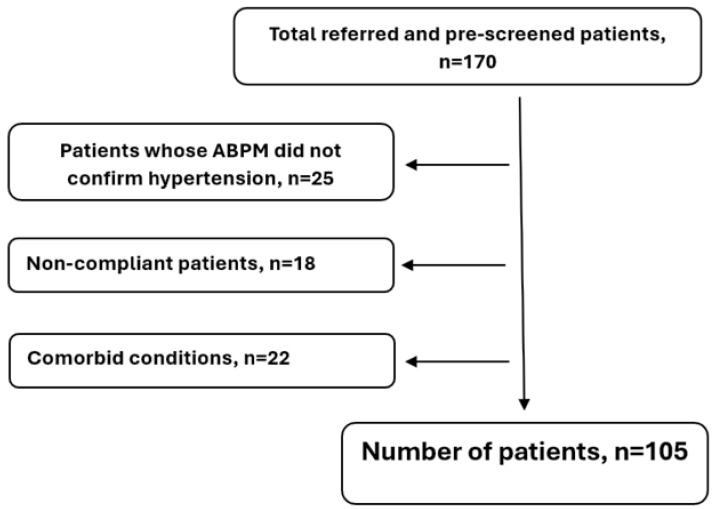
Exclusion process.

The exclusion criteria included previous stroke, TIA, poor general condition, a life expectancy of <5 years, and comorbidities that significantly influenced this study: diabetes, severe heart disease, arrhythmia, renal failure, tumors, significant carotid artery stenosis, autoimmune disease, psychiatric disorders, dementia, etc. A “silent” infarction or other organic abnormalities detected in cranial CT also resulted in exclusion. Additional exclusion criteria included pregnancy, postpartum period, alcohol dependence, and extreme obesity, which was defined as a body mass index (BMI) of greater than 35 kg/m^2^, as this level is associated with significant comorbidities and health risks. The exclusion process is presented in Figure 1.

### 2.1. Methods

**Figure 2:**
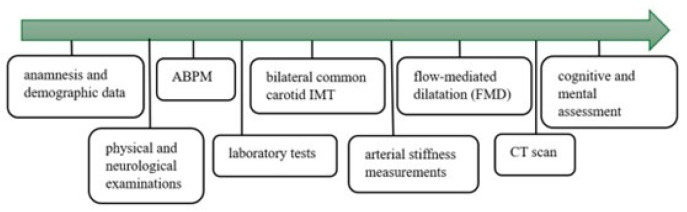
Study protocol.

The available data covered detailed medical history, demographic variables, the results of physical and neurological examinations, cognitive and mental assessment, and laboratory tests, including serum electrolytes, renal function, glucose, lipid profiles, complete blood count, CRP, hemoglobin A1C, fibrinogen levels, and urinalysis. The study protocol is summarized in Figure 2.

These assessments were followed by 24 h ambulatory blood pressure monitoring (ABPM). Blood pressure was measured every 15 min during the day (from 6:00 to 22:00) and every 30 min at night (from 22:00 to 6:00). Based on the ABPM data, an internal medicine specialist evaluated the results, determined daytime and nighttime mean systolic and diastolic blood pressures, and the systolic and diastolic hyperbaric index. For the ABPM measurements, Cardiospy ABPM equipment from Labtech Ltd. (Debrecen, Hungary, model: EC-ABP) was used.

The bilateral common carotid IMT was measured with a 7.5 MHz linear probe (Philips HD 11 XE from Unicorp Biotech Kft., Budapest, Hungary). The IMT was measured on the far wall of the common carotid artery [15].

Flow-mediated dilatation (FMD) measurement of the brachial artery was performed using an HP Sonos 5500 ultrasound device with a 10 MHz linear test transducer (from National Utrasound, Tampa, FL, USA). The FMD was expressed as the percentage increase in the resting diameter of the artery after cuff release (the forearm cuff was inflated to suprasystolic pressure for 5 min, 10–40 mmHg above the patient’s systolic pressure [16]. The brachial artery FMD is a marker of endothelial damage in large arteries [17].

Arterial stiffness measurements were performed using a TensioClinic arteriograph (TensioMed Ltd., Budapest, Hungary). Arterial stiffness was assessed by determining the augmentation index (AIx) and pulse wave velocity (PWV) [18]; the latter measures the rate of pulse wave propagation between the carotid and femoral arteries, which mainly reflects the thickening of the tunica media [19]. Abnormal stiffness and FMD values reflect the likelihood of future vascular injury, and their values are interrelated [20].

### 2.2. Neuropsychological Evaluation

All patients underwent a detailed 90 min long neuropsychological evaluation with the guidance of a psychologist. The test was designed to determine the main neurocognitive functions listed in the 5th Edition of the *Diagnostic and Statistical Manual of Mental Disorders* (DSM-5): reaction time, attention, executive function, learning, memory, and perceptual-motor skills. The test package included the most sensitive tests to reveal minor differences in cognitive function that are not necessarily detectable during everyday activities. For the statistical analysis, all variables were standardized, inverse scales were reversed, and all were summed as total.

The order of tasks was different from subject to subject to control for the effect of the task order on performance (e.g., due to fatigue). Two key aspects were considered when the sequence of tasks was created. Exercises requiring creativity (e.g., the five-point test and verbal fluency tests) were performed prior to those measuring intelligence. During the 20 min delay of the delayed recognition task, only nonverbal tasks were performed to avoid interference. Before the baseline assessment, we drew randomly from these tests. All neurocognitive functions were measured, especially the most sensitive ones to hypertension, which are attention, executive functions, and memory. The assessed cognitive domains and the validated tests used are shown in Table 1.

### 2.3. Statistical Methods

The main characteristics of the patients were described with means ± SD in the case of continuous variables, and proportions were calculated for categorical variables. The chi-squared test was used to assess the relationship between the categorical variables. The Shapiro–Wilk test was used to assess normality. Because of the non-normal distribution of the variables, medians and interquartile ranges (IQRs) were calculated. Spearman’s correlation was used to estimate the extent of correlations between continuous variables. Correlation coefficients were interpreted based on Akoglu’s work [39]. A composite measure (cognitive index) was created, which summarized the achievements of a person. Some indicators were reversed, and others were standardized to eliminate the differences in dimensions and to have a straight tendency. The cut-off value for the FMD/IMT ratio was found by cyclic linear regression models based on possible integer values, and we performed ROC analysis. Multiple linear regression models were created to evaluate the associations, which were found in simple analyses, and to adjust for potential confounders. Microsoft Excel (Microsoft Excel, 2016) and the Intercooled Stata v13.0 software were used for the data analysis. To interpret the Spearman’s correlation coefficient, cut-off points generally accepted in medicine were used [39].

## 3. Results

A total of 105 subjects were enrolled in this study (Table 2), with a mean (±standard deviation) age of 45.08 ± 11.42 years and a male-to-female ratio of 2.09. The systolic and diastolic mean ABPM results (± standard deviation) were 144.37 ± 10.96 and 87.14 ± 7.58 mmHg.

The descriptive statistics of the blood test results and the ABPM, IMT, FMD, AIX, and PWV parameters are in the Appendix A and Appendix B. The descriptive statistics of the cognitive test results are in Table 3.

To combine the effects of the most important variables, a composite index was derived as the ratio between the FMD value and the mean of the left and right carotid IMT values. This indicator was created to neutralize the effect of between-patient differences in physique: a carotid of greater diameter is inherently paired with a greater IMT; thus, the differences between thinner and thicker vessels are eliminated.

In the first step, the cognitive domains correlated with the FMD were identified.

The FMD showed a fair (rho = 0.39) positive significant (*p* = 0.007) correlation with working memory, a poor (rho = 0.29) positive significant (*p* = 0.016) correlation with complex attention, a fair (rho = 0.34) positive significant (*p* = 0.005) correlation with learning abilities, and a fair (rho = 0.39) positive significant (*p* = 0.014) correlation with executive functions. These results are presented in Table 4. It can be concluded that with decreasing FMD values, performance in working memory, attention, learning abilities, and executive functions also deteriorates.

In the second stage, the cognitive domains related to the IMT were found.

The IMT showed a fair (rho = −0.49) negative significant (*p* < 0.001) correlation with complex attention and a fair (rho = −0.42) negative significant (*p* < 0.001) correlation with reaction time. These results are presented in Table 4. Thus, as the IMT values increase, reaction time decreases, attention deteriorates, and the efficiency of executive functions worsens.

Next, we examined the relationship between the derived FMD/IMT ratio and the overall index generated from the results of cognitive function assessments.

**Figure 3:**
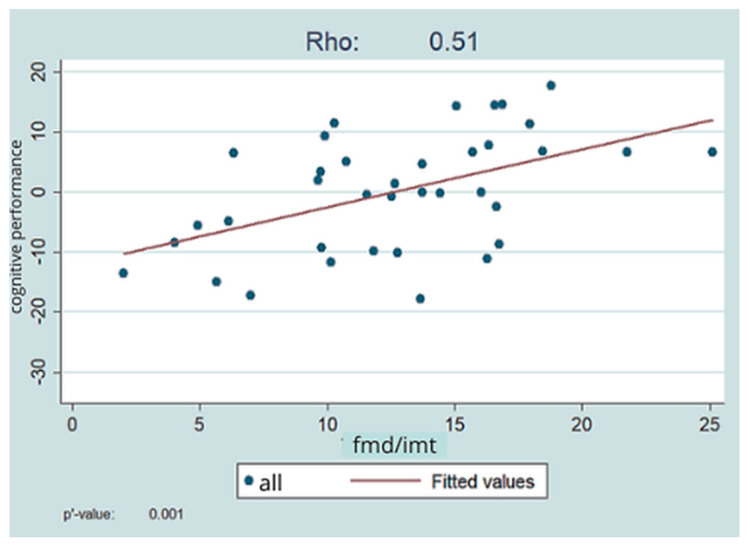
Relationship between the FMD/IMT ratio and the overall cognitive index.

The composite FMD/IMT value showed a fair (rho = 0.51) positive significant (*p* = 0.001) relationship with the overall index of cognitive functions, i.e., better FMD and IMT values were associated with better cognitive performance. This is shown in Figure 3.

Examining the relationship between the FMD/IMT ratio and the overall cognitive index, we looked at which FMD/IMT value can be used as a cut-off for cognitive impairment. Based on our results, an FMD/IMT ratio of 15 represents a cut-off value (*p* = 0.002; R-squared = 0.23).

This indicates that below a cut-off value of 15 FMD/IMT, it is worth initiating a detailed cognitive assessment, and below FMD and IMT values of 6.44 and 0.43, respectively, deterioration of working memory, complex attention, reaction time, learning, and executive functions are expected.

**Figure 4:**
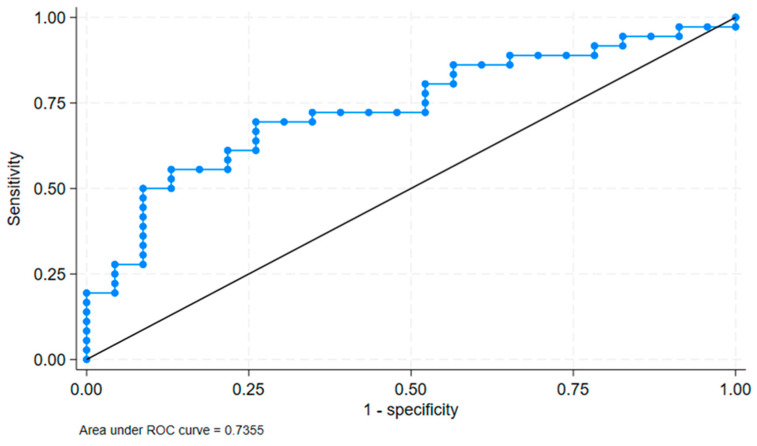
Results of ROC analysis.

We verified the cut-off value with ROC analysis. The ROC analysis showed an area under the curve (AUC) of 0.7355 with a standard error of 0.0658 and a 95% confidence interval of [0.60655, 0.86446]. This is shows in Figure 4. This indicates a good discriminatory ability of the FMD/IMT binary variable at a cut-off of 14.47 to distinguish between the presence and absence of cognitive impairment.

AIx and PWV measured by arteriography did not show significant correlations with the overall index of cognitive function. As for the PWV, a significant (*p* = 0.010) fair (rho = −0.31) negative correlation was seen with working memory and a significant (*p* = 0.012) poor (rho = −0.27) negative correlation with complex attention. In the case of AIx, a fair negative (rho = −0.36) significant (*p* < 0.001) relationship was detected with reaction time and a significant (*p* = 0.020) fair (rho = −0.30) negative relationship with executive functions. The significant findings are presented in Table 5.

We performed a regression analysis with covariatee adjustment, and in the case of FMD/IMT, the significant positive association remained (0.2314 [0.0198–0.443]) after adjusting for age group and gender. The findings are presented in Table 6.

## 4. Discussion

The most important new observation of our study is that by combining the results of two rapid tests (IMT and FMD), we obtained a cut-off value that helps in the selection of hypertensive individuals for whom time-consuming, complex cognitive tests are expected to produce abnormal results. In patients with FMD/IMT ratios lower than a cut-off of 15, we will most likely find cognitive differences. These are expected in the domains of reaction time, attention, executive functions, and working memory.

Hypertension is a chronic disease that causes multiorgan damage (arteries, heart, kidney, and central nervous system) over time [40]. Untreated hypertension also impairs cognition [41]. As hypertension simultaneously damages large blood vessels (e.g., carotid and brachial), there has been a need in the past for easily and rapidly detectable vascular markers (IMT, FMD, and stiffness) that serve as biomarkers to screen individuals at increased risk for vascular events. Flow-mediated dilatation (FMD), carotid intima–media thickness (IMT), and arterial stiffness (pulse wave velocity (PWV) and augmentation index (AIX)) are sensitive, non-invasive parameters used to measure the severity of endothelial function and vascular wall damage [42]. Previous studies have described that carotid intima–media thickening and arterial stiffness are markers of arteriosclerosis [43,44]. The results of a 2020 study confirmed the association between intima–media thickness and cognitive decline and the notion that an increase in intima–media thickness predicts the subsequent development of cognitive decline was also supported. Memory functions and attention were the most commonly affected domains [45]. Csipo and colleagues demonstrated by FMD and stiffness studies that poorer FMD and stiffness levels are associated with poorer cognitive performance in the elderly [46]. Others have observed an association between stiffness and memory impairment in men. Based on their results, increased arterial stiffness was significantly and independently associated with memory impairment in the men they studied [47].

A detailed review of the association between stiffness and cognitive function was also published; it found an association between pathological stiffness and cognitive decline [48]. In our own study, we found no significant association between cognitive differences and stiffness, which may be explained by the fact that our study population consisted of relatively young individuals (mid-40s) whose hypertension had presumably persisted for a short time. In their study, Kearney-Schwartz et al. observed a significant positive correlation between memory scores and PWV in males [47]. The results of our own study did not support between-gender differences; however, in the above-mentioned study, the mean age was more than 20 years older than in our own study group, and patients with chronic hypertension were studied, while our research involved newly diagnosed patients. In a study of patients with minor neurocognitive impairment, Tonacci et al. found decreased FMD values [49]. Tachibana et al. also measured decreased levels of FMD in vascular dementia patients compared with healthy controls. This study considered FMD to be a more sensitive marker than IMT [50]. A report on 10 studies processing data from 2791 patients found that impairment of FMD is associated with poorer neuropsychological performance, particularly in executive functions and working memory tasks [51]. In hypertensive patients studied by Saka et al., FMD was significantly decreased and inversely correlated with intima–media thickness as well as diastolic blood pressure. They suggested that carotid IMT is a possible indicator of abnormal endothelial function based on a positive correlation with hypertension and a significant negative correlation with FMD [52]. According to Smith et al., impaired FMD is associated with poorer neurocognitive function in overweight, hypertensive patients [53]. Del Brutto et al. highlight the role of age in the analysis of the relationship between IMT and cognitive functions [45]. Our own study did not yield similar results in terms of age, but we emphasize that we studied relatively young, asymptomatic, newly diagnosed patients, whereas in Del Gross’s study, the mean age was more than 10 years older. In a 2016 review, Naiberg et al. [51] summarized the results of studies examining the relationship between FMD and neurocognition. They concluded that studies to date suggest impaired FMD being most strongly associated with attention, executive function, and working memory tasks. Our own study also showed a significant correlation with impaired levels of FMD in relation to learning, which is a novel finding in the literature.

Another main goal of our study was to determine which cognitive domains are primarily impaired in early hypertension. With this, our goal was to find a select group of sensitive tests that are likely to be abnormal to be used instead of time-consuming detailed cognitive tests. To our knowledge, there has never been a study that has examined the relationships between cognitive function and the parameters presented above for a similar purpose. In their 2009 study, Kearney-Schwartz et al. [47] found significant memory impairment in hypertensive patients. We did not detect memory impairment but found impaired complex attention, response time, working memory, and executive functions. The reason for the difference between these findings may have been the younger age of our patients, as Kearney-Schwartz studied older patients who were already expected to have memory impairment. The results of Naiberg et al., in turn, confirm our observations, as they also found early impairment of attention, executive functions, and working memory [51]. Deterioration of executive functions and psychomotor speed was also observed by Smith et al. [53].

This study had several limitations that should be acknowledged. This work identified a composite vascular marker (FMD/IMT ratio) to screen for cognitive impairment in young hypertension patients, providing a useful tool for early management. The single-center study methodology and limited sample size may restrict the generalizability of the findings. To confirm the findings, more research, including older and more varied groups, is required. Furthermore, the cross-sectional design precludes the evaluation of cognitive and vascular changes over time and prevents causal inferences. Longitudinal studies are needed to establish causal relationships and the temporal progression of cognitive decline in hypertension. Excluding persons with concomitant illnesses like diabetes and obesity may not accurately represent the hypertensive population. Future research should include patients with these comorbidities to determine the generalizability of our findings. Future research should also aim for a larger, more diverse population, with long follow-ups to validate the findings and assess the impact of pressure-reducing therapy on cognitive outcomes.

## 5. Conclusions

In our study, we investigated the relationships between physiological parameters associated with hypertension and cognitive function in newly diagnosed, young, primary hypertensive patients.

For the FMD/IMT ratio, a cut-off value was determined to identify patients for whom early cognitive function assessment is of paramount importance. In addition, we confirmed an association between increased intima–media thickness and lesser performance in complex attention and response time and between reduced flow-mediated dilatation and poorer attention, learning, working memory, and executive functions.

Based on our results, IMT and FMD levels and their composite ratio are suitable for the identification of particularly vulnerable patients who require detailed cognitive examination for the early detection and treatment of functional disorders. Although significant, the moderate correlation coefficients indicate that FMD and IMT are part of a multifactorial framework affecting cognitive function. Future research should investigate additional factors to improve the accuracy of risk stratification.

In early hypertension, assessment is required of the cognitive domains of attention, executive functions, working memory, and reaction time. The examination and monitoring of performance in these cognitive domains is important in hypertensive individuals who rely on such performance in their daily work.

Our findings demonstrate that the FMD/IMT ratio is a useful and sensitive predictor of early cognitive impairment in young hypertension patients. However, additional multicenter research with larger populations and longer follow-ups would be helpful to verify these findings. Future studies will investigate whether early interventions, such as cognitive training, lifestyle changes, and improved hypertension management based on FMD and IMT values, can mitigate cognitive decline, thereby justifying their use as routine markers in clinical practice.

## Figures and Tables

**Table 1:** The cognitive domains assessed and the tests used for their evaluation.

Cognitive Domains and Functions Examined	Neuropsychological Assessment Battery
Reaction time	
Reaction time	Computerized reaction time assessment
Complex attention	
Psychomotor speed Visuomotor coordination Processing speed	Digit Symbol Substitution Test [21,22]
Attention	Toulouse–Piéron test [23,24]
Processing speed Selective attention, cognitive flexibility	Victoria Stroop Test [25,26]
Executive functions	
Inhibition	Victoria Stroop Test [25,26]
Switching, cognitive flexibility	Trail Making Test [27]
Visual fluency, cognitive flexibility	Five-Point Test [28,29,30]
Executive function	Digit Span Test [31,32]
Working memory	
Spatial–visual working memory	Corsi block-tapping test [33,34]
Verbal working memory	Digit Span Test (backward) [31,32]
Learning and memory	
Immediate verbal memory	Rey-AVLT [34,35,36]
Language	
Verbal fluency	Verbal fluency test: letter, category, and action fluency [37,38]
Perceptual-motor skills	
Psychomotor speed Visuomotor coordination, synthesis Visual perception and construction	WAIS Mosaic Test [21,22]
Cognitive domains and functions examined	Neuropsychological assessment battery

**Table 2:** Descriptive statistics of age and BMI in the sample.

Variable	*n*	Mean ± SD	Median [IQR]
Age	105	45.08 ± 11.42	44.00 [39.00–51.00]
BMI	103	28.60 ± 4.79	27.80 [25.60–31.20]

**Table 3:** Descriptive statistics of cognitive function indices.

Variable	*n*	Mean ± SD	Median [IQR]
Working memory	70	27.67 ± 4.50	28.00 [25.00–31.00]
Language	95	96.59 ± 21.50	97.00 [84.00–111.00]
Complex attention	93	0.03 ± 2.12	0.29 [−1.01–1.71]
Perceptual-motor skills	96	11.89 ± 2.89	13.00 [10.00–14.00]
Reaction time	92	−1.23 ± 0.18	1.21 [−1.36–1.10]
Learning	96	62.86 ± 11.85	64.00 [55.50–72.00]
Executive functions	60	−0.02 ± 3.01	0.55 [−0.99–1.82]
Overall cognitive index	59	0.68 ± 10.30	2.84 [−5.56–7.21]

**Table 4:** Significant correlations between IMT or FMD and performance in various cognitive domains.

	Rho	Significance (*p*)
FMD–working memory	0.31	0.044
FMD–attention	0.27	0.029
FMD–learning	0.35	0.004
FMD–executive functions	0.34	0.037
IMT–complex attention	−0.49	<0.001
IMT–reaction time	−0.42	<0.001

**Table 5:** Cognitive domains significantly associated with PWV and AIx values.

	Rho	Significance (*p*)
PWV–working memory	−0.31	0.010
PWV–complex attention	−0.27	0.012
AIX–reaction time	−0.36	<0.001
AIX–executive functions	−0.30	0.020

**Table 6:** Regression analysis with adjustment of covariates.

	FMD	IMT	FMD/IMT
All	0.124 [0.029–0.219]	−0.002 [−0.007–0.002]	0.249 [0.072–0.426]
Age group (45+/18–45)	0.859 [−1.095–2.814]	0.1 [0.022–0.179]	−1.17 [−4.585–2.244]
Gender (female/male)	−0.291 [−2.199–1.617]	−0.035 [−0.117–0.048]	0.829 [−2.797–4.455]
FMD		−0.003 [−0.019–0.013]	
IMT	−1.507 [−9.759–6.745]		

## Data Availability

The data that support the findings of this study are available from the corresponding author upon reasonable request. The data are not publicly available due to containing health and medical information that could compromise the privacy of the research participants.

## Appendix A

**Table A1:** Descriptive statistics of blood test results.

Variable	*n*	Mean ± SD	Median [IQR]	Interval
Na mmol/L	85	140.41 ± 2.18	141 [139–142]	133–146
K mmol/L	85	4.27 ± 0.31	4.2 [4.1–4.5]	3.5–5.3
Cl mmol/L	83	104.08 ± 2.22	104 [103–106]	99–111
Glucose mmol/L	87	5.3 ± 1.15	5 [4.7–5.6]	3.6–6.0
Urea mmol/L	86	4.99 ± 1.27	4.9 [4.1–5.8]	2.8–8.0
Creatinine imol/L	87	76.34 ± 15.68	75 [66–88]	44–84
GOT U/L	85	23.71 ± 9.54	21 [18–26]	<40
GGT U/L	87	43.34 ± 60.02	28 [19–43]	7–50
Fibrinogen g/L	81	3.17 ± 0.96	3.08 [2.49–3.48]	1.5–4
TGs mmol/L	87	1.81 ± 2.2	1.3 [0.8–1.9]	<1.7
Chol mmol/L	87	5.16 ± 1	5 [4.6–5.6]	2.0–5.2
HDL-C mmol/L	84	1.3 ± 0.38	1.29 [1–1.5]	>1.3
LDL-C mmol/L	83	3.33 ± 0.79	3.2 [2.89–3.7]	1.0–3.4
HgbA1c mmol/mol	67	5.35 ± 0.66	5.3 [5–5.6]	4.2–6.1
ESR mm/h	82	11.51 ± 7.31	12 [6–16]	0–15
WBC G/L	88	6.63 ± 1.62	6.58 [5.63–7.26]	4.5–10.8
RBC T/L	88	4.93 ± 0.4	4.92 [4.67–5.17]	4.2–5.4
Hgb g/L	88	145.9 ± 13.09	146 [139–154.5]	115–150
Htc	88	0.43 ± 0.03	0.43 [0.41–0.45]	0.35–0.47
CRP mg/L	32	4.77 ± 5.69	2.35 [1.3–6.2]	<4.6
Thr G/L	86	240.77 ± 60.21	221 [205–275]	150–400

Na = sodium, K = calcium, Cl = chloride, GOT = glutamic-oxaloacetic transaminase, GGT = gamma-glutamyl transferase, TGs = triglycerides, Chol = cholesterol, HDL-C = high-density lipoprotein cholesterol, LDL-C = low-density lipoprotein cholesterol, HgbA1c = hemoglobin A1c, ESR = erythrocyte sedimentation rate, WBC = white blood cell, RBC = red blood cell, Hgb = hemoglobin, Htc = hematocrit, CRP = C-reactive protein, and Thr = thrombocyte.

## Appendix B

**Table A2:** Descriptive statistics of ABPM, IMT, FMD, AIX, and PWV parameters.

Variable	*n*	Mean ± SD	Median [IQR]
left_carotid_imt_lcca	92	0.59 ± 0.12	0.58 [0.51–0.66]
right_carotid_imt_rcca	92	0.57 ± 0.11	0.54 [0.49–0.61]
carotid_mean_imt_rcca_lcca	82	0.58 ± 0.11	0.56 [0.5–0.62]
fmd_dilation	66	7.67 ± 3.04	7.54 [5.75–9.2]
fmd/imt	58	14.32 ± 6.71	13.78 [9.79–17.31]
arteriograph_aix	93	–15.78 ± 33.08	−16.2 [−43.9–10.4]
arteriograph_pwv	93	9.99 ± 2.29	9.7 [8.6–11.1]
abpm_active_sbp	92	147.87 ± 11.11	147 [141.5–152]
abpm_active_dbp	92	88.76 ± 11.25	89 [84–93.5]
abpm_active_shi	90	384.08 ± 174.76	355 [266–439]
abpm_active_dhi	90	221.39 ± 98.26	212 [158–265]
abpm_active_pp	92	57.93 ± 7.81	58 [51.5–63]
abpm_passive_sbp	90	129.88 ± 18.2	129 [121–140]
abpm_passive_dbp	90	77.74 ± 8.99	76 [71–84]
abpm_passive_shi	88	392.9 ± 250.75	328 [226.5–529.5]
abpm_passive_dhi	88	193.09 ± 155.82	164 [86–262.5]
abpm_passive_pp	90	52.89 ± 9.36	51.5 [47–58]
abpm_mean_sbp	93	144.37 ± 10.96	143 [139–150]
abpm_mean_dbp	93	87.14 ± 7.58	86 [82–91]
abpm_mean_shi	90	391.78 ± 179.08	365 [275–451]
abpm_mean_dhi	90	226.32 ± 93.56	215.5 [161–272]
abpm_mean_pp	92	56.87 ± 7.84	57 [50–62]
abpm_mean_map	48	198.29 ± 114.39	163 [107.5–248]

imt = intima–media thickness, lcca = left common carotid artery, rcca = right common carotid artery, fmd = flow-mediated dilatation, aix = augmentation index, pwv = pulse wave velocity, abpm = ambulatory blood pressure monitoring, active = day, passive = night, mean = 24 h, and sbp = systolic blood pressure: measures the pressure in your arteries when your heart beats. dbp = diastolic blood pressure: measures the pressure in your arteries when your heart rests between beats. shi = systolic hyperbaric index (or hyperbaric area) is defined as the area above the reference systolic blood pressure (upper normal limit) in the 24 h systolic blood pressure curve. dhi = diastolic hyperbaric index (or hyperbaric area) is defined as the area above the reference diastolic blood pressure (upper normal limit) in the 24 h diastolic blood pressure curve. pp = pulse pressure is defined as the difference between peak systolic blood pressure and the end-diastolic blood pressure. map = mean arterial pressure is defined as the average pressure in a patient’s arteries during one cardiac cycle. To calculate the mean arterial pressure, double the diastolic blood pressure, and add the sum to the systolic blood pressure (JCS 2010).
